# Supplementary material for: Modeling glioblastoma heterogeneity as a dynamic network of cell states
Source: Mol Syst Biol. 2021 Sep 16;17(9):e10105. doi: 10.15252/msb.202010105 (PMC8444284; doi:10.15252/msb.202010105)
Supplement: Supplementary file 6 — Source Data for Figure 5 [file MSB-17-e10105-s004.zip › Figure5A_sourcedata/GSEA_3071/nosigdb_stateV.GseaPreranked.1622795159954/RICHARDS_NEFTEL_CELL_2019_MES1.html]

Details for gene set RICHARDS\_NEFTEL\_CELL\_2019\_MES1[GSEA]

|  || Dataset | state43071 |
| Phenotype | NoPhenotypeAvailable |
| Upregulated in class | na\_neg |
| GeneSet | RICHARDS\_NEFTEL\_CELL\_2019\_MES1 |
| Enrichment Score (ES) | -0.7073435 |
| Normalized Enrichment Score (NES) | -3.1976104 |
| Nominal p-value | 0.0 |
| FDR q-value | 0.0 |
| FWER p-Value | 0.0 |
Table: GSEA Results Summary

  

Fig 1: Enrichment plot: RICHARDS\_NEFTEL\_CELL\_2019\_MES1      
 Profile of the Running ES Score & Positions of GeneSet Members on the Rank Ordered List

  

| PROBE | GENE SYMBOL | GENE\_TITLE | RANK IN GENE LIST | RANK METRIC SCORE | RUNNING ES | CORE ENRICHMENT || 1 | A2M |  |  | 532 | 0.846 | -0.0783 | No |
| 2 | C1R |  |  | 1696 | 0.437 | -0.2876 | No |
| 3 | WWTR1 |  |  | 1794 | 0.419 | -0.2947 | No |
| 4 | S100A16 |  |  | 1824 | 0.414 | -0.2890 | No |
| 5 | ANXA1 |  |  | 2165 | 0.366 | -0.3438 | No |
| 6 | GJA1 |  |  | 3602 | -0.316 | -0.6084 | No |
| 7 | GSN |  |  | 3695 | -0.331 | -0.6169 | No |
| 8 | VIM |  |  | 3830 | -0.354 | -0.6328 | No |
| 9 | TUBA1C |  |  | 3957 | -0.379 | -0.6465 | No |
| 10 | CLIC4 |  |  | 4075 | -0.404 | -0.6578 | No |
| 11 | APOE |  |  | 4229 | -0.449 | -0.6748 | No |
| 12 | PLP2 |  |  | 4366 | -0.490 | -0.6874 | No |
| 13 | IFITM3 |  |  | 4424 | -0.509 | -0.6844 | No |
| 14 | MT1X |  |  | 4481 | -0.530 | -0.6807 | No |
| 15 | IGFBP7 |  |  | 4622 | -0.604 | -0.6910 | Yes |
| 16 | TAGLN2 |  |  | 4648 | -0.618 | -0.6790 | Yes |
| 17 | ACTN1 |  |  | 4775 | -0.695 | -0.6841 | Yes |
| 18 | LGALS1 |  |  | 4784 | -0.699 | -0.6667 | Yes |
| 19 | MT2A |  |  | 4799 | -0.714 | -0.6500 | Yes |
| 20 | NPC2 |  |  | 4841 | -0.750 | -0.6375 | Yes |
| 21 | GFPT2 |  |  | 4873 | -0.780 | -0.6222 | Yes |
| 22 | CLIC1 |  |  | 4899 | -0.809 | -0.6050 | Yes |
| 23 | NAMPT |  |  | 4906 | -0.820 | -0.5840 | Yes |
| 24 | NNMT |  |  | 5004 | -0.942 | -0.5769 | Yes |
| 25 | CTSB |  |  | 5007 | -0.948 | -0.5516 | Yes |
| 26 | S100A10 |  |  | 5011 | -0.954 | -0.5263 | Yes |
| 27 | EMP1 |  |  | 5023 | -0.979 | -0.5018 | Yes |
| 28 | LGALS3 |  |  | 5039 | -1.017 | -0.4771 | Yes |
| 29 | ANXA2 |  |  | 5055 | -1.036 | -0.4519 | Yes |
| 30 | EMP3 |  |  | 5084 | -1.113 | -0.4271 | Yes |
| 31 | TIMP1 |  |  | 5134 | -1.251 | -0.4025 | Yes |
| 32 | MT1E |  |  | 5154 | -1.323 | -0.3702 | Yes |
| 33 | SERPINE1 |  |  | 5161 | -1.377 | -0.3340 | Yes |
| 34 | S100A11 |  |  | 5196 | -1.536 | -0.2989 | Yes |
| 35 | EFEMP1 |  |  | 5199 | -1.550 | -0.2572 | Yes |
| 36 | MGP |  |  | 5252 | -2.037 | -0.2119 | Yes |
| 37 | SPP1 |  |  | 5272 | -2.446 | -0.1492 | Yes |
| 38 | MGST1 |  |  | 5286 | -2.788 | -0.0762 | Yes |
| 39 | FN1 |  |  | 5287 | -2.866 | 0.0015 | Yes |
Table: GSEA details [plain text format]

  

Fig 2: RICHARDS\_NEFTEL\_CELL\_2019\_MES1: Random ES distribution      
 Gene set null distribution of ES for **RICHARDS\_NEFTEL\_CELL\_2019\_MES1**
